# Fast modal decomposition for optical fibers using digital holography

**DOI:** 10.1038/s41598-017-06974-7

**Published:** 2017-07-26

**Authors:** Meng Lyu, Zhiquan Lin, Guowei Li, Guohai Situ

**Affiliations:** 1 Shanghai Institute of Optics and Fine Mechanics, Chinese Academy of Sciences, Shanghai, 201800 China; 2 University of Chinese Academy of Sciences, Beijing, 100049 China

## Abstract

Eigenmode decomposition of the light field at the output end of optical fibers can provide fundamental insights into the nature of electromagnetic-wave propagation through the fibers. Here we present a fast and complete modal decomposition technique for step-index optical fibers. The proposed technique employs digital holography to measure the light field at the output end of the multimode optical fiber, and utilizes the modal orthonormal property of the basis modes to calculate the modal coefficients of each mode. Optical experiments were carried out to demonstrate the proposed decomposition technique, showing that this approach is fast, accurate and cost-effective.

## Introduction

Modal decomposition (MD) of the field at the output end of the optical fiber can provide fundamental insights into the nature of electromagnetic-wave propagation through it, because if the underlying modes that make up the optical field are known, all the physical quantities associated with the field, e.g., intensity, phase, wavefront, beam quality factor, Poynting vector and orbital angular momentum density can be inferred^1^. Thus, MD techniques have been widely used in many optical applications, such as laser mode competition and oscillations^2^, bend loss^3^, optimizing fiber-to-fiber coupling^4^, and the analysis of multimode^5–9^ and large mode area (LMA)^10–14^ fibers. In particular, LMA fibers have been widely used in industrial processing, defence industry and scientific applications because large core diameters of LMA fibers can suppress nonlinear effects so as to obtain high output power and high beam quality^15^. However, as the core diameter increases, it becomes difficult to maintain strictly transverse single mode operation. As a consequence, LMA-fiber-based high power fiber laser systems are usually not strictly single mode^16^. Meanwhile, mode instability in high-power fiber lasers occurs when the laser power exceeds a certain threshold^17,18^. Thus, it is technically necessary to analyze the mode component for the step-index LMA fiber in order to achieve quasi-single mode beam^19^. One conventional way to characterize beam quality is to measure the *M*^2^ parameter, which is based on the beam waist width and divergence angle^20^. However, the *M*^2^ parameter does not provide detailed quantitative information about the mode content and may give misleading information about the beam quality of step-index LMA fibers^16^. Therefore, MD techniques are also highly desired in designing and optimizing step-index LMA fiber structures^14^.

In the past few years, various MD techniques have been proposed. The underlying theory of all these techniques is that an optical field in a fiber can be decomposed into a series of orthogonal linear modes, each weighted with a complex coefficient^21^. In 2004, Soh *et al*. have developed a theory to decompose the modes based on the retrieval of the mutual intensity profile, and uses the idea of lateral shearing interferometry to decompose the modal contents^5^. This wavefront measurement technique consists of analyzing the interference pattern generated by several replicas of the incoming beam that are propagating at different angles^13^ which requires very precise alignment. Iterative phase retrieval algorithms have been adopted for MD as well. The technique utilizes the mapping of the two-dimensional electric field onto a one-dimensional space of waveguide eigenmodes, and employs the Gerchberg-Saxton algorithm to extract the amplitudes and the phases of all the guided modes^6^. Multidimensional optimization algorithms such as the Nelder-Mead simplex algorithm and the stochastic parallel gradient descent algorithm have also been introduced to analyze few-mode fibers^7,8,10^. However, it is well known that these are not convex optimization algorithms, and may end up with local minima. Recently, Nicholson *et al*. have demonstrated an MD technique based on both spatially and spectrally (S^2^) resolving the image of the output of the fiber under test^14^. Later on, Nguyen *et al*. have improved the S^2^ technique and made it more accurate, simpler, and faster^9^. This technique can provide high quality images of the modes. However, it relies on the measurement of the four-dimensional spectrogram of the beam, which requires raster scanning in the transverse directions, in a way similar to the measurement of phase space distribution function^22^. The other powerful MD method is to used a computer-generated hologram (CGH) as the matched filter in a 4f correlator^11^. The CGH is designed by interfering an ideal mode pattern with a reference beam. A central peak is formed at the output plane when the incoming beam has a mode that matches the hologram. Otherwise, a central null is formed. The CGHs used in this case are usually fabricated lithographically, which is costly and inflexible in terms of reconfiguration. As such, spatial light modulator (SLM) is a good substitute for the lithographic CGH^12^.

In this paper, we propose a novel complete MD technique for step-index optical fibers based on the principle of digital holography (DH). As examples of demonstration, we use it to analyze the modes for a step-index communication fiber and a LMA fiber. The proposed technique acquires the electric field at the output end of an optical fiber by DH, and calculates the modal weights and the relative phases of all modes by virtue of the orthonormal property. We show that the proposed technique has three advantages. First, the optical setup is very simple, and reconfigurable. It does not require transverse scanning of the beam. Second, it requires only one 2D intensity measurement, instead of the 4D spectrogram, to analyze the mode components. And finally, the proposed MD method is deterministic, does not require any optimization algorithm to find the weighting parameters. These allow it to work very fast, with the time only limited by the frame rate of the camera. It thus has great potential in the characterization of step-index fibers.

## Results

**Figure 1:**
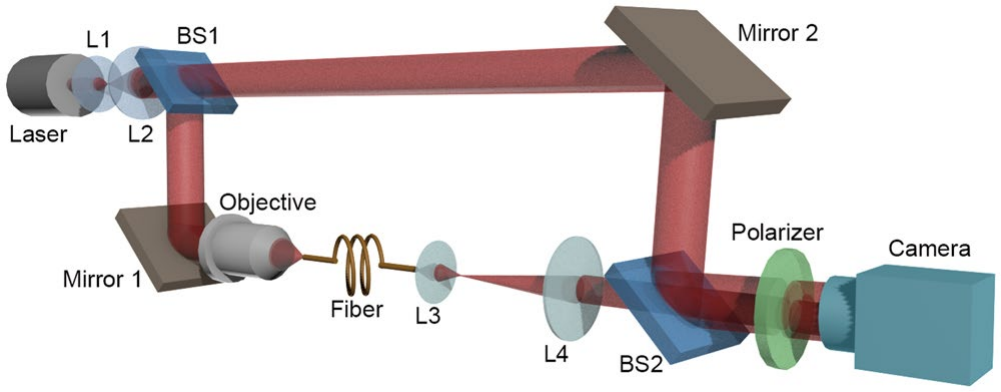
Experimental setup for MD experiments, BS1 and BS2 are beam splitters, L1, L2, L3 and L4 are lenses.

The optical setup used in our experiments are schematically illustrated in Fig. 1, which is a typical off-axis digital holography architecture. A collimated and expanded He-Ne laser beam was split into two by a beam splitter (BS1). One part was coupled into the fiber under analysis using a microscopic objective and thus called object beam, denoted by ${U}_{\perp }^{^{\prime} }(x,y)$. A 4f imaging system consisting of a suitable lens L3 and lens L4 projected the emitting electric field at the output end of the fiber normally onto a image sensor. The other part was combined with the object beam by the second beam splitter (BS2), serving as the reference beam, which can be written as *R*(*x*, *y*) = *r*(*x*, *y*) exp (−*ikx* sin *θ*), where *r*(*x*, *y*) is the magnitude, *λ* represents the wavelength and *k* = 2*π*/*λ* is the wave number of the beam. The angle between the reference and the object beam, *θ*, can be tuned by using the mirror 2 and BS2. A linear polarizer was placed in front of the image sensor to select the intended polarization state. What the image sensor captured was actually the interference pattern produced by the object and reference beams

1 $$\begin{array}{rcl}{I}_{H}(x,y) & = & {|R(x,y)+{U}_{\perp }^{^{\prime} }(x,y)|}^{2}=r{(x,y)}^{2}+{|{U}_{\perp }^{^{\prime} }(x,y)|}^{2}\\  &  & +r(x,y)\exp (-ikx\,\sin \,\theta ){U}_{\perp }^{^{\prime} }{(x,y)}^{\ast }\\  &  & +r(x,y)\exp (ikx\,\sin \,\theta ){U}_{\perp }^{^{\prime} }(x,y),\end{array}$$

where the asterisk denotes the complex conjugate. The first two terms of the Eq. (1) are the 0th order terms, and the last two terms carry the information about the object wavefront. And these two terms can be separated in the Fourier spectrum when the reference beam is adjusted to some suitable angles^23^. Thus one can use a band-pass filter to select the term with ${U}_{\perp }^{^{\prime} }(x,y)$, and eventually reconstruct the whole wavefront of the object. Owing to this capability, DH has been widely used in many fields such as industrial^24^ and biological inspections^25^, imaging through complex media^26^, and fiber measurements^27^, just to name a few.

In the following subsections, we will show the results of mode decomposition of a step-index communication fiber and a LMA fiber.

### SMF-28 communication fiber

The first fiber we tested in our experiment was an typical SMF-28 communication fiber with the core diameter *a* = 8.2 *μ*m, and the numerical aperture NA = 0.14. This fiber was designed as a single mode fiber operating in the infrared region (*λ* = 632.991 nm in our experimental setup). However, multiple modes can be excited when a laser beam in the visible spectral region is coupled in. Once the core radius *a*, the numerical aperture of the fiber and the wavelength *λ* of the propagating light are specified, all the eigenmodes *Ψ*_*mn*_ (*x*, *y*) that the fiber can support are determined. Thus, we can calculate their modal coefficients *c*_*k*_ and the modal weights *ρ*_*k*_. One can refer to the Methods Section for the theoretical description.

According to the parameters, it is easy to calculate that the communication fiber can support the following 10 eigenmodes: *LP*_01_, *LP*_02_, *LP*_11*e*_, *LP*_11*o*_, *LP*_12*e*_, *LP*_12*o*_, *LP*_21*e*_, *LP*_21*o*_, *LP*_31*e*_ and *LP*_31*o*_, where *e* and *o* represent the even and the odd mode, respectively.

**Figure 2:**
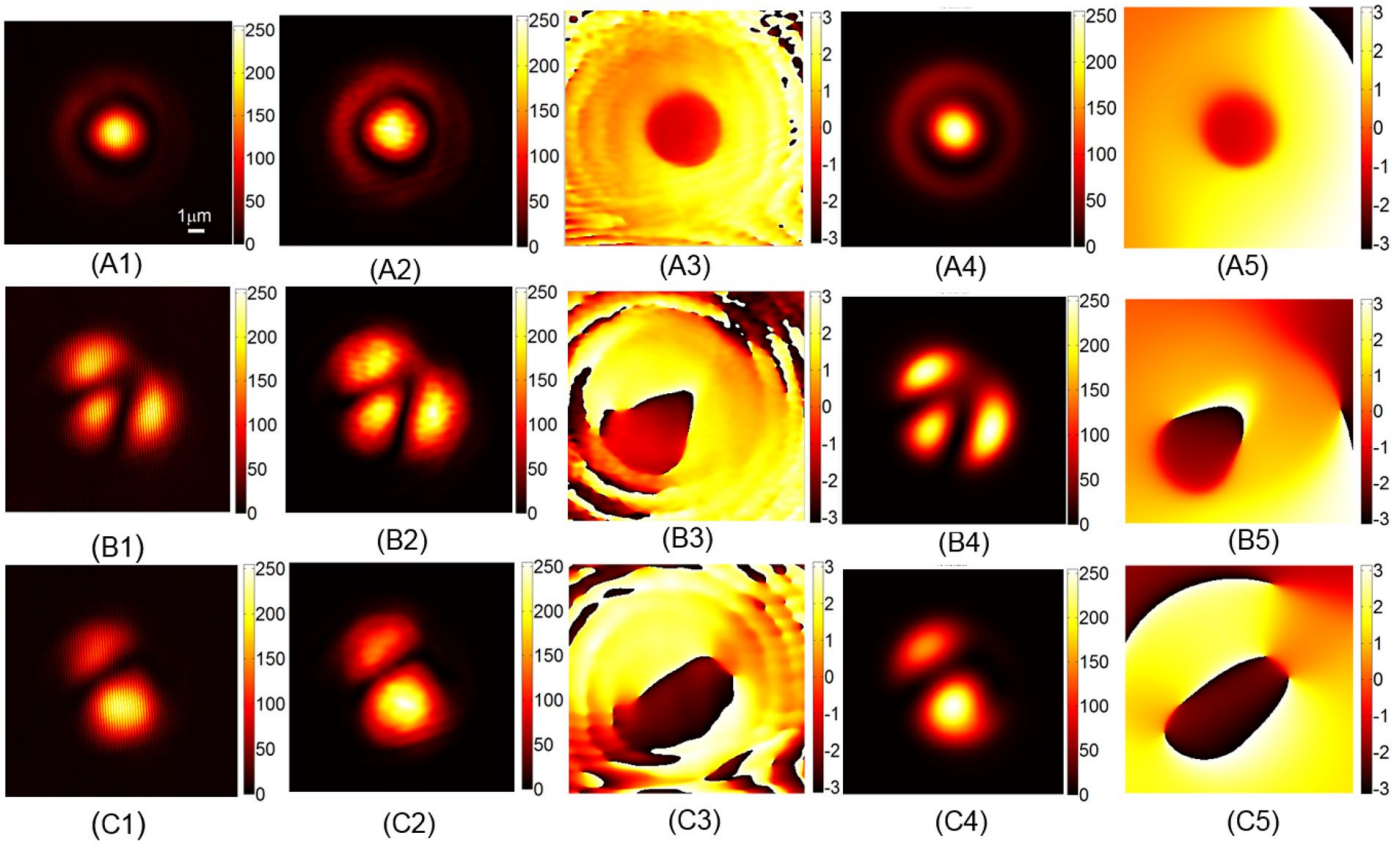
Modal decomposition results of the communication fiber. (**A1**,**B1** and **C1**) The holograms recorded by the camera. The ratio of the average power density between the object beams and the reference beams is about 5:1. (**A2**,**B2** and **C2**) The intensity patterns reconstructed from the holograms. (**A3**,**B3** and **C3**) The phase maps reconstructed from the holograms. (**A4**,**B4** and **C4**) The intensity patterns synthesized by modal coefficients. (**A5**,**B5** and **C5**) The synthesized phase maps.

**Figure 3:**
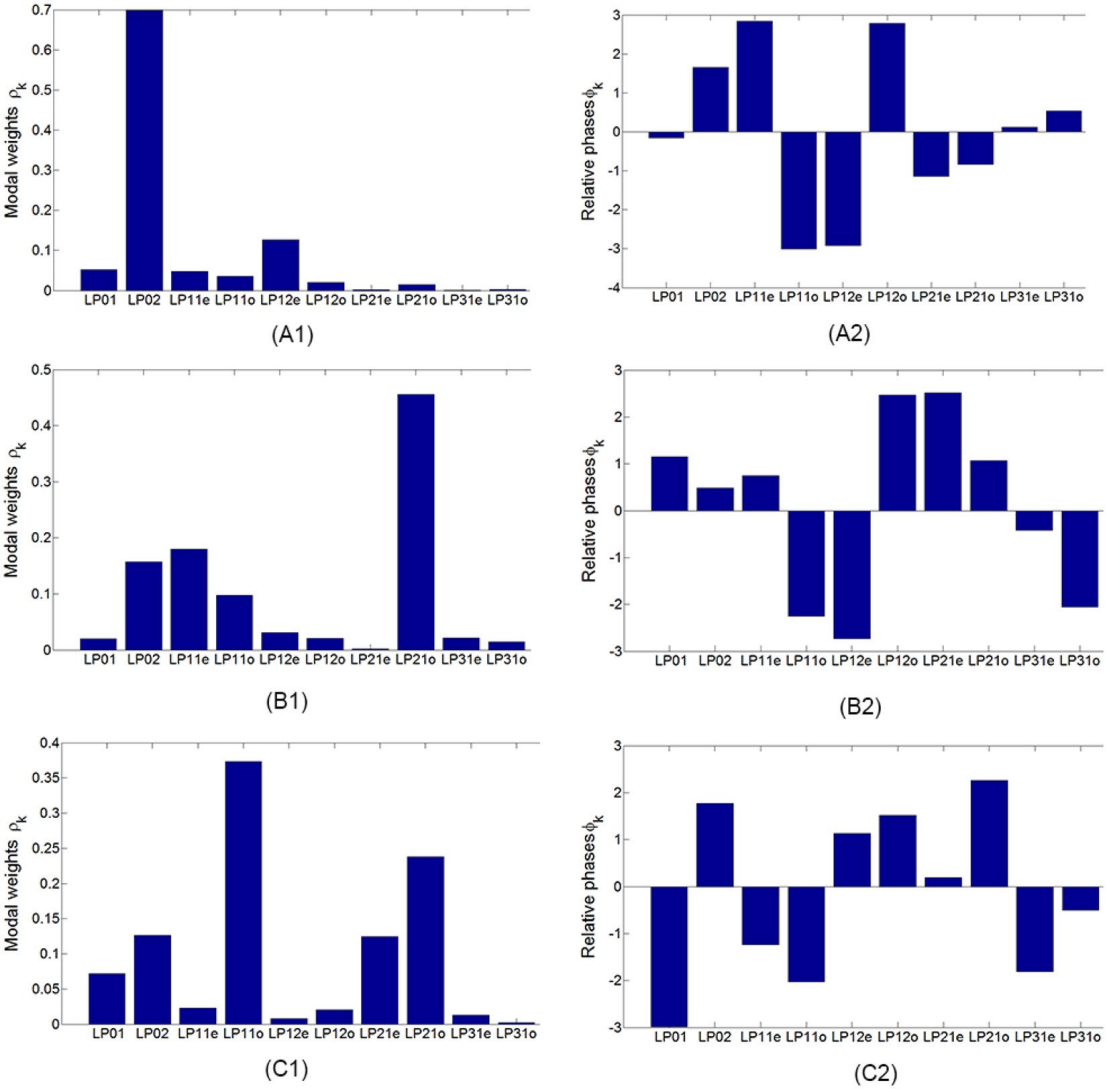
The modal coefficients of the communication fiber. (**A1**,**B1** and **C1**) The modal weights *ρ*_*k*_. (**A2**,**B2** and **C2**) The relative phases *ϕ*_*k*_.

The main results are plotted in Figs 2 and 3. For the demonstration, we changed the initial condition of the coupling of the laser into the fiber, which was realized by tuning the relative position between the microscopic objective and the fiber. In this way, we observed three different patterns at the output end of the fiber. The digital holograms of these electric fields are plotted in Fig. 2A1,B1 and C1, respectively. The ratio of the average power density between the object beams and the reference beams is about 5:1. With these holograms at hand, we reconstructed the whole wavefront of the electric fields, the intensity patterns and the phases of which are plotted in Fig. 2A2,B2,C2,A3,B3 and C3, respectively. Then the complex modal coefficients ${c}_{k}={\alpha }_{k}\exp (i{\varphi }_{k})$, where *k* = 1, …, 10, can be calculated directly from the reconstructed wavefront using the orthonormal property (See Methods for details), and the results are plotted in Fig. 3. The modal weights *ρ*_*k*_ = |*α*_*k*_|^2^ for all the 10 base modes associated with the three patterns are plotted in Fig. 3A1,B1 and C1, and their relative phases *ϕ*_*k*_ in Fig. 3A2,B2 and C2, respectively.

In order to verify the new MD technique, we synthesized the three electric fields using the modal coefficients *c*_*k*_ plotted in Fig. 3. The intensity patterns of the synthesized fields are shown in Fig. 2A4,B4 and C4, and the corresponding phase maps in Fig. 2A5,B5 and C5, respectively. It is clearly seen from Fig. 2 that the synthesized intensity patterns are highly similar to those holographically reconstructed shown in Fig. 2A2,B2 and C2 respectively. So do the phase maps, as comparing the figures in the fifth and the third columns of Fig. 2. To quantify the similarity, we calculated the correlation coefficients *γ* between the synthesized patterns from the modal coefficients and the reconstructed ones from the acquired holograms

2 $$\gamma =\frac{\sum ({I}_{rec}-{\bar{I}}_{rec})({I}_{syn}-{\bar{I}}_{syn})}{\sqrt{\sum {({I}_{rec}-{\bar{I}}_{rec})}^{2}}\sqrt{\sum {({I}_{syn}-{\bar{I}}_{syn})}^{2}}},$$

where *I*_*syn*_ and *I*_*rec*_ are the intensity patterns of the synthesized and the reconstructed light fields, respectively, and ${\bar{I}}_{syn}$ and ${\bar{I}}_{ret}$ are their means. In the experiments, the correlation coefficients between the three synthesized and the corresponding intensity patterns are 0.9860, 0.9649 and 0.9835, respectively, suggesting that the electric field outcomes from the step-index communication fiber can be exactly decomposed into base modes characterized by the modal coefficients *c*_*k*_.

### LMA fiber

**Figure 4:**
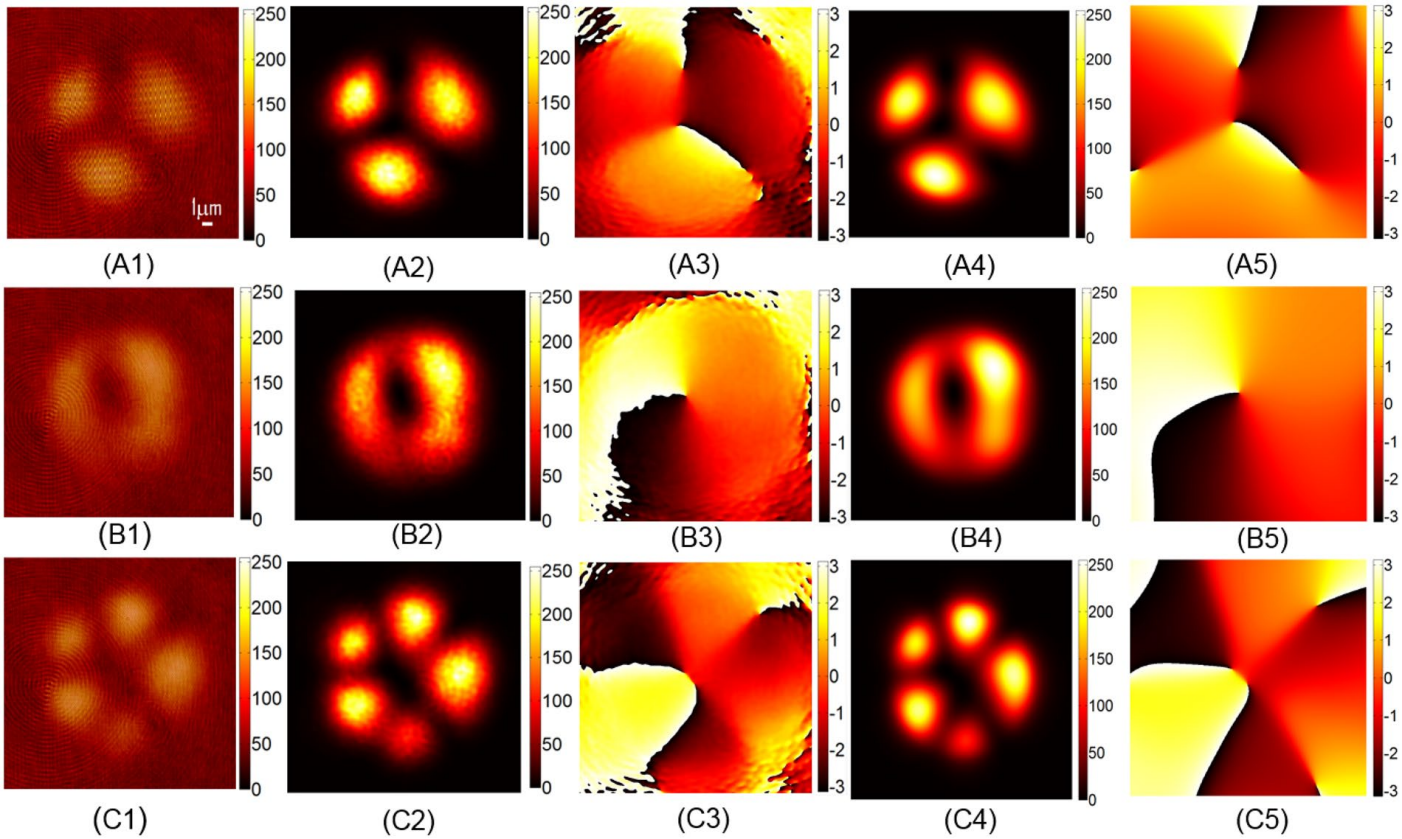
Modal decomposition results of the LMA fiber. (**A1**,**B1** and **C1**) The holograms recorded by the camera. The ratio of the average power density between the object beams and the reference beams is about 1:1. (**A2**,**B2** and **C2**) The intensity patterns reconstructed from the holograms. (**A3**,**B3** and **C3**) The phase maps reconstructed from the holograms. (**A4**,**B4** and **C4**), The intensity patterns synthesized by modal coefficients. (**A5**,**B5** and **C5**) The synthesized phase maps.

The step-index LMA fiber we used to test our technique had a core diameter *a* = 20 *μ*m, and the numerical aperture NA = 0.065. Accordingly, the LMA fiber can support the following 12 eigenmodes: *LP*_01_, *LP*_02_, *LP*_11*e*_, *LP*_11*o*_, *LP*_12*e*_, *LP*_12*o*_, *LP*_21*e*_, *LP*_21*o*_, *LP*_31*e*_, *LP*_31*o*_, *LP*_41*e*_ and *LP*_41*o*_, where *e* and *o* represent the even and the odd mode, respectively. Compared with the SMF-28 fiber, the modes of LMA fiber only add two modes: *LP*_41*e*_ and *LP*_41*o*_, because the LMA fiber has a low NA. Because of this, the light was coupled into the LMA fiber through a lens with the focal length *f* = 200 mm in this case. Due to the improvement of the coupling efficiency, the power density of object beam is increased. Thus we replaced the 50:50 (R:T) BS1 in the SMF-28 fiber experiments with a 10:90 (R:T) beamsplitter cube in order to balance the power density between the object beam and the reference beam. The resulting ratio of the average power density between the object beams and the reference beams is about 1:1 as shown in Fig. 4A1,B1 and C1.

**Figure 5:**
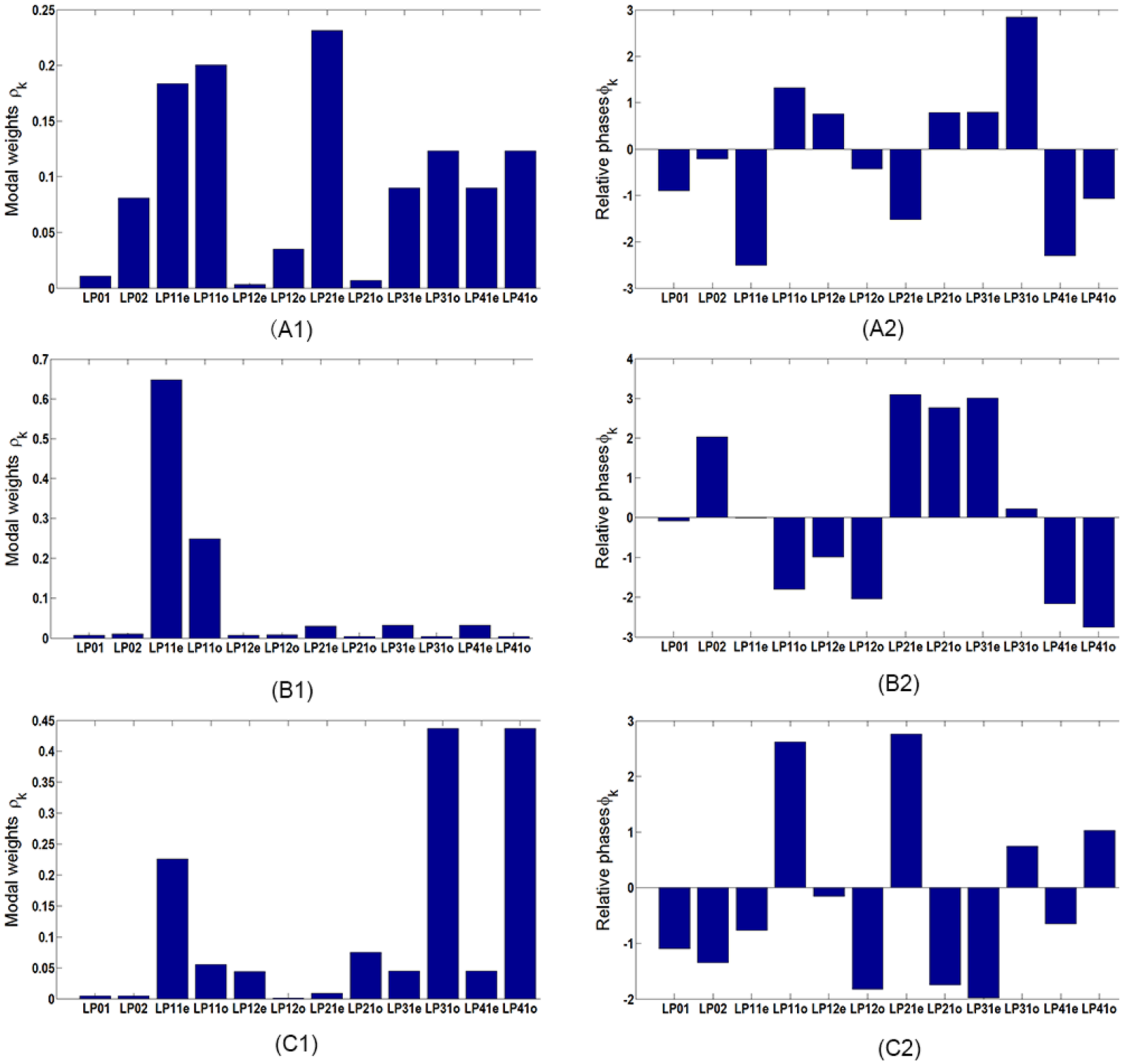
The modal coefficients of the LMA fiber. (**A1**,**B1** and **C1**) The modal weights *ρ*_*k*_. (**A2**,**B2** and **C2**) The relative phases *ϕ*_*k*_.

Again, we tuned the coupling condition and obtained three different patterns at the output end of the LMA fiber. With their holograms recorded by the camera (shown in Fig. 4(A1,B1 and C1), respectively), we reconstructed the whole wavefront of the LMA fiber from the three holograms, the intensity and the phase patterns of which are shown in Fig. 4(A2,B2,C2,A3,B3 and C3), respectively. The complex modal coefficients *c*_*k*_ were calculated directly from the reconstructed wavefronts. The modal weights *ρ* and relative phase *ϕ* of *c*_*k*_ are plotted in the left and the right column, respectively, in Fig. 5. Again, we synthesized the three electric fields using the modal coefficients *c*_*k*_, and the intensity patterns and the phase maps of the synthesized fields are shown in Fig. 4(A4,B4,C4,A5,B5 and C5), respectively. The correlation coefficients between the synthesized intensity patterns from the modal coefficients and the reconstructed ones from the holograms are 0.9937, 0.9955 and 0.9788, respectively, indicating that the electric field outcomes from the step-index LMA fiber can be faithfully decomposed into the base modes characterized by the modal coefficients *c*_*k*_.

## Discussion

So far we have demonstrated experimentally an novel MD method for optical fibers utilizing DH. The experimental results are in excellent agreement with theoretical prediction. Now we summarize the strength of the proposed method. First, as an interferometric technique, the proposed DH-based MD method is sensitive to change of power of the higher-order-modes supported by the fiber, allowing the decomposition of the modes with high confidence. Second, the proposed MD technique employs the off-axis geometry, and needs only one intensity measurement. Compared to other MD techniques that require multiple intensity measurements, the proposed one can have higher time efficiency. Third, the proposed MD technique is a non-iterative method, which means that it needs less time to process the acquired experimental data, and without any risk of falling into the local minimum. Due to these reasons, the proposed MD technique has the potential to operate at frame rate speed, in particular when high frame-rate camera and the graphic processing unit are integrated into the system.

One can see that there are phase residuals in the reconstructed field as shown in the third column in both Figs 2 and 4. This may due to the defocus of the either the signal beam or the reference beam. However, the modes propagates in free space independently, and will not interact with one another. Such slight defocus will not affect the results of the mode decomposition. One can see Supplementary Information.

It is worthy of pointing out that we considered only one polarization state of the electric field coming out from the fiber in our experiments. For a more general configuration, two orthogonal LP modes should be taken. To achieve this, we can successively measure two holograms for two orthogonal polarizations components of the electric field by rotating the polarizer in Fig. 1 or holographically record the two orthogonal polarization components in a single hologram by using polarization multiplexing techniques^28^. To obtain better results, we can reduce the aberrations and improve the resolution of the imaging system. In the setup, the main imaging system is a 4f system consisting of two lenses: L3 and L4. If we use a microscopic objective and a tube lens instead of aspheric lens L3 and lens L4, aberrations of the imaging system can be reduced significantly. Specifically in the setup shown in Fig. 1, the sizes of the imaging on the camera sensor are about 140 × 140 pixels for the communication fiber and about 441 × 441 pixels for the LMA fiber and it utilizes less than 3.21% pixels of the camera sensor. A higher magnification imaging system can take full advantage of the camera pixels and result in much better performance.

## Methods

### Modal decomposition theory

In accordance with the optical waveguide theory, arbitrary electric field in step-index optical fibers satisfying the assumption of weak guiding can be described as a superposition of linear polarized (LP) eigenmodes, each of which is weighted with a complex expansion coefficient, namely, the modal coefficient *c*_*k*_^21^. Note that there are two polarization states in each LP mode, namely the x- and y-polarized states, and these two sets of polarizations are mutually perpendicular in space. Hence, we can consider only one polarization at a time without loss of generality. Then an arbitrary y-polarized light field *U*_⊥_(*x*, *y*) in the fiber is regarded as

3 $${U}_{\perp }(x,y)=\sum _{k=1}^{{k}_{\max }}{c}_{k}{\Psi }_{mn}(x,y)$$

where *Ψ*_*mn*_ (*x*, *y*) is the transverse eigenmode structure inside the core, and (*x*, *y*) denotes the transverse Cartesian coordinates.

Due to the orthonormal property of fibers^11,21,29^,

4 $$\begin{array}{l}\langle {\Psi }_{i},{\Psi }_{j}\rangle ={\iint }_{{R}^{2}}{\Psi }_{i}(x,y){\Psi }_{j}^{\ast }(x,y){\rm{d}}x{\rm{d}}y={\delta }_{ij}(x,y)=\{\begin{array}{ll}1, & i=j\\ 0, & others\end{array}\end{array}$$

where the asterisk denotes the complex conjugate, the modal coefficients *c*_*k*_ can be calculated by

5 $${c}_{k}={\alpha }_{k}\exp (i{\varphi }_{k})=\langle {U}_{\perp },{\Psi }_{mn}\rangle ={\iint }_{{R}^{2}}{U}_{\perp }(x,y){\Psi }_{mn}^{\ast }(x,y)dx{\rm{d}}y,$$

the modal weights *ρ*_*k*_ = |*c*_*k*_|^2^ = |*α*_*k*_|^2^ fulfilling the relation ∑|*c*_*k*_|^2^ = ∑*ρ*_*k*_ = 1, and *ϕ*_*k*_ represents the relative phases.

### Experimental parameters

In the experiments, the laser was a linearly polarized He-Ne laser (Thorlabs, Inc. HRS015) irradiates at 632.991 nm. The BS2 is a 50:50 non-polarizing beamsplitter cube. L3 is an aspheric lens with the focal length *f* = 2 mm and the NA is 0.5, and L4 is a lens with *f* = 200 mm. These two lenses made a 100× 4f imaging system.

For the step-index communication fiber, the BS1 is a 50:50 (R:T) non-polarizing beamsplitter cube. The microscopic objective is a 10× plan achromat objective and its NA is equal to 0.25. The recording camera is a CMOS camera (Point Grey, GS3-U3–23S6M-C) with the maximum pixel count of 1920 × 1200 and pixel size of 5.86 *μ*m × 5.86 *μ*m. The tested optical fiber was a SMF-28 fiber which is a single-mode fiber at the wavelength of 1310 nm but becomes multimode at the wavelength of 632.991 nm. Its NA is 0.14, the core diameter is *a* = 8.2 *μ*m, the cladding diameter is 125.0 ± 0.7 *μ*m and the length is about 10 m.

For the step-index LMA fiber, the BS1 is a 10:90 (R:T) non-polarizing beamsplitter cube. The microscopic objective was replaced by a lens with focal length *f* = 200 mm and the diameter *d* = 25.4 mm in order to match the low NA of the LMA fiber. The recording camera was a CCD camera (Allied Vision Technologies, GX2750) with the maximum pixel count of 2750 × 2200 and pixel size of 4.54 *μ*m × 4.54 *μ*m. The tested optical fiber is a typical LMA fiber which is a multimode fiber at the wavelength of 632.991 nm. Its NA is 0.065, the core diameter is *a* = 20 *μ*m, the cladding diameter is 400*μ* m and the length is about 5 m.

### Off-axis DH

**Figure 6:**
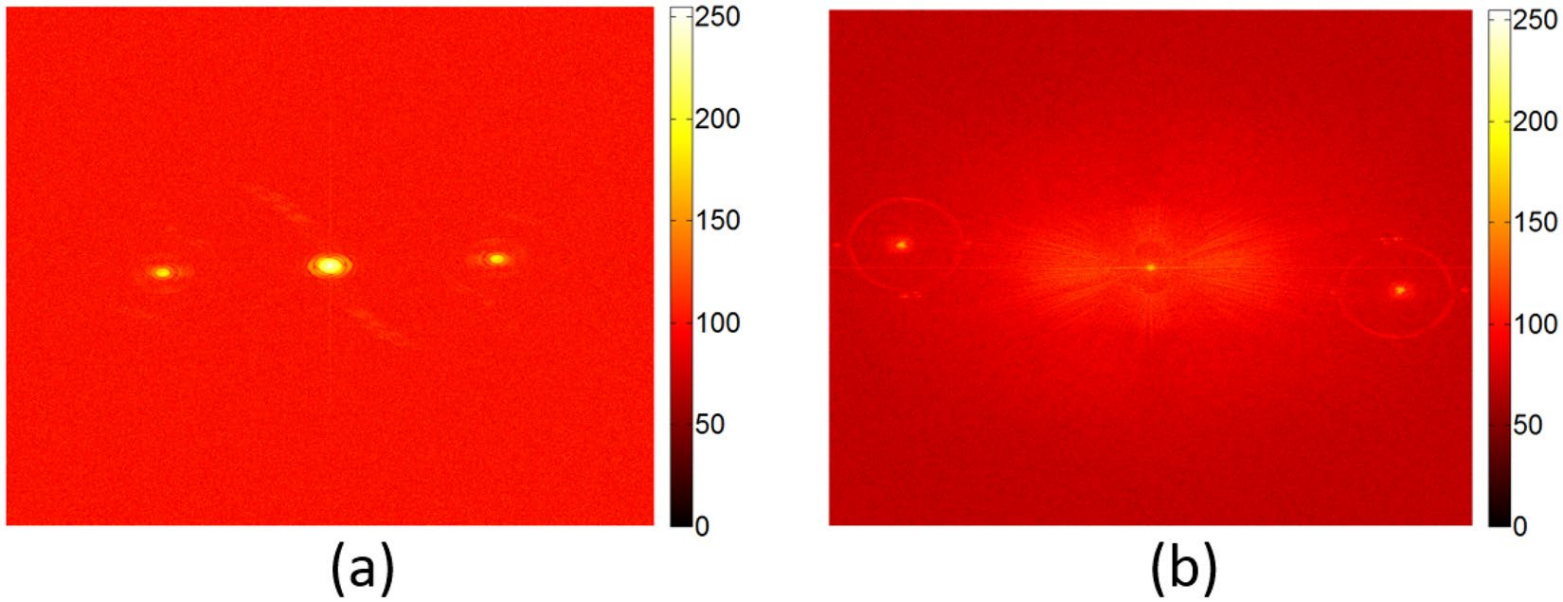
The Fourier spectrum of two holograms. (**a**) The Fourier spectrum of Fig. 2A1. (**b**) The Fourier spectrum of Fig. 4A1.

Note that the Fourier transform of Eq. (1), we have the spectrum of the recorded hologram

6 $$ {\mathcal F} [{I}_{H}(x,y)]={G}_{z}({f}_{x},{f}_{y})+r{G}_{t}^{\ast }({f}_{x}+\frac{k\,\sin \,\theta }{2\pi },{f}_{y})+r{G}_{t}({f}_{x}-\frac{k\,\sin \,\theta }{2\pi },{f}_{y})$$

where we have taken the reference beam *r* being a plane wave into account in our experiment, ${\mathcal F}$ represents two-dimensional Fourier transform, *G*_*z*_ denotes the zeroth-order component, and *G*_*t*_ represents the first order of the spectrum, respectively. In order to separate the zeroth order and the two twin images, we aligned the tilted angle of the reference beam so that the relation $\sin (\theta )\ge 3B\lambda$, where *B* is the bandwidth of *G*_*t*_ held. Specifically, the angle *θ* is 1.767° for the communication fiber and 3.096° for the LMA fiber in our experiments. Figure 6 plots the Fourier spectrum of two typical holograms for the SMF-28 fiber and the LMA fiber we tested, respectively. Obviously, the zeroth-order and the two twin images are well separated. Then, the third term $r{G}_{t}({f}_{x}-k\,\sin \,\theta /2\pi ,{f}_{y})$ in Eq. (6) is extracted by a two dimensional Butterworth filter with the radius of 150 pixels and the center located at (*k* sin *θ*/2*π*, 0). Finally, the wavefront $r{U}_{\perp }^{^{\prime} }(x,y)$ was obtained by the inverse Fourier transform of the filtered spectrum followed by the multiplication of the phase factor exp (−*ikx* sin *θ*).

### Data post-processing

#### SMF-28 fiber

It is easy to calculate the normalized frequency of the communication fiber we used is *V* = *ak*_0_*NA* = 5.6975. Then, a set of modes that our fiber can support can be calculated by using the characteristic equation of the fiber^11,21^. The values of *U* and *W* associated with these LP modes are listed in Table 1.

**Table 1:** The values of *U* and *W* for the LP modes that the communication fiber used for the demonstration can support.

Modes	*LP*_01_	*LP*_02_	*LP*_11*e*_, *LP*_11*o*_	*LP*_12*e*_, *LP*_12*o*_	*LP*_21*e*_, *LP*_21*o*_	*LP*_31*e*_, *LP*_31*o*_
*U*	2.0386	4.5839	3.2311	5.6275	4.3000	5.2872
*W*	5.3223	3.3868	4.6950	0.9019	3.7407	2.1279

As the 4f imaging system has a magnification of 100 and the pixel size of CMOS sensor is 5.86 *μ*m × 5.86 *μ*m, the sampling interval at the output surface of the fiber is 5.86/100 = 0.0586 *μ*m. As such, the sampling interval of the eigenmodes *Ψ*_*mn*_ (*x*, *y*) is also 0.0586 *μ*m. According to the specification, the core diameter of the fiber we used is 8.2 *μ*m. It is easy to calculate the fiber core can be sampled to be 140 × 140. In the experiments, we zero-padding it to be 300 × 300. According to Table 1, we generated all the eigenmodes *Ψ*_*mn*_ (*x*, *y*) from the transverse eigenmode structures and characteristic equation with the same sampling condition^11,21^, and calculated their modal coefficients *c*_*k*_ and the modal weights *ρ*_*k*_ according to Eq. (4). Considering the experimental and numerical error, we finely adjusted the center position and the sampling interval of the eigenmodes *Ψ*_*mn*_ (*x*, *y*) until the correlation coefficients *γ* is maximum.

#### LMA fiber

The normalized frequency of the LMA fiber we used is *V* = 6.4520, and the values of *U* and *W* associated with these LP modes are listed in Table 2. Because the LMA fiber and the communication fiber are step-index fibers, the data post-processing method of the LMA fiber is the same as the communication fiber.

**Table 2:** The values of *U* and *W* for the LP modes that the LMA fiber used for the demonstration can support.

Modes	*LP*_01_	*LP*_02_	*LP*_11*e*_, *LP*_11*o*_	*LP*_12*e*_, *LP*_12*o*_	*LP*_21*e*_, *LP*_21*o*_	*LP*_31*e*_, *LP*_31*o*_	*LP*_41*e*_, *LP*_41*o*_
*U*	2.0768	4.7025	3.2969	5.8773	4.3978	5.4294	6.3977
*W*	6.1086	4.4176	5.5461	2.6619	4.7210	3.4857	0.8355

## Electronic supplementary material

Supplementary Information

## References

[1] Schulze C, Ngcobo S, Duparré M, Forbes A (2012). Modal decomposition without a priori scale information. Opt. Express.

[2] Napartovich AP, Vysotsky DV (2007). Theory of spatial mode competition in a fiber amplifier. Phys. Rev. A.

[3] Schermer RT, Cole JH (2007). Improved bend loss formula verified for optical fiber by simulation and experiment. IEEE J. Quantum Electron.

[4] Flamm D (2013). Modal characterization of fiber-to-fiber coupling processes. Opt. Lett..

[5] Soh DBS (2004). Modal power decomposition of beam intensity profiles into linearly polarized modes of multimode optical fibers. J. Opt. Soc. Am. A.

[6] Shapira O, Abouraddy AF, Joannopoulos JD, Fink Y (2005). Complete modal decomposition for optical waveguides. Phys. Rev. Lett..

[7] Lü H, Zhou P, Wang X, Jiang Z (2013). Fast and accurate modal decomposition of multimode fiber based on stochastic parallel gradient descent algorithm. Appl. Opt..

[8] Huang L (2015). Real-time mode decomposition for few-mode fiber based on numerical method. Opt. Express.

[9] Nguyen DM (2012). Modal decomposition technique for multimode fibers. Appl. Opt..

[10] Stutzki F (2011). High-speed modal decomposition of mode instabilities in high-power fiber lasers. Opt. Lett..

[11] Kaiser T, Flamm D, Schröter S, Duparré M (2009). Complete modal decomposition for optical fibers using CGH-based correlation filters. Opt. Express.

[12] Flamm D, Naidoo D, Schulze C, Forbes A, Duparré M (2012). Mode analysis with a spatial light modulator as a correlation filter. Opt. Lett..

[13] Paurisse M, Lévèque L, Hanna M, Druon F, Georges P (2012). Complete measurement of fiber modal content by wavefront analysis. Opt. Express.

[14] Nicholson J, Yablon A, Ramachandran S, Ghalmi S (2008). Spatially and spectrally resolved imaging of modal content in large-mode-area fibers. Opt. Express.

[15] Jauregui C, Limpert J, Tünnermann A (2013). High-power fibre lasers. Nat. Photon..

[16] Wielandy S (2007). Implications of higher-order mode content in large mode area fibers with good beam quality. Opt. Express.

[17] Eidam T (2011). Experimental observations of the threshold-like onset of mode instabilities in high power fiber amplifiers. Opt. Express.

[18] Eidam T (2010). Femtosecond fiber CPA system emitting 830 w average output power. Opt. Lett.

[19] Kong F (2016). Large-mode-area fibers operating near single-mode regime. Opt. Express.

[20] Zervas MN, Codemard CA (2014). High power fiber lasers: a review. IEEE J. Sel. Top. Quantum Electron..

[21] Snyder, A. W. & Love, J. *Optical Waveguide Theory* (Springer Science & Business Media, 2012).

[22] Waller L, Situ G, Fleischer JW (2012). Phase-space measurement and coherence synthesis of optical beams. Nat. Photon..

[23] Situ G, Sheridan JT (2007). Holography: an interpretation from the phase-space point of view. Opt. Lett..

[24] Tian L, Loomis N, Domnguez-Caballero JA, Barbastathis G (2010). Quantitative measurement of size and three-dimensional position of fast-moving bubbles in air-water mixture flows using digital holography. Appl. Opt..

[25] Cotte Y (2013). Marker-free phase nanoscopy. Nat. Photon..

[26] Zhang Y (2013). Application of short-coherence lensless Fourier-transform digital holography in imaging through diffusive medium. Opt. Commun..

[27] Colomb T (2005). Polarization microscopy by use of digital holography: application to optical-fiber birefringence measurements. Appl. Opt..

[28] Yuan C, Situ G, Pedrini G, Ma J, Osten W (2011). Resolution improvement in digital holography by angular and polarization multiplexing. Appl. Opt..

[29] Amnon, Y. & Yeh, P. *Optical Eelectronics in Modern Communications* (Oxford University Press, 1997).

