# Author Correction: Fast modal decomposition for optical fibers using digital holography

**DOI:** 10.1038/s41598-018-23448-6

**Published:** 2018-03-22

**Authors:** Meng Lyu, Zhiquan Lin, Guowei Li, Guohai Situ

**Affiliations:** 1 Shanghai Institute of Optics and Fine Mechanics, Chinese Academy of Sciences, Shanghai, 201800 China; 2 University of Chinese Academy of Sciences, Beijing, 100049 China

Correction to: *Scientific Reports* 10.1038/s41598-017-06974-7, published online 26 July 2017

The original version of this Article contained an error in Affiliation 2, which was incorrectly given as ‘University of the Chinese Academy of Sciences, Beijing, 100049, China’. The correct affiliation is listed below:

University of Chinese Academy of Sciences, Beijing, 100049, China.

This error has now been corrected in the HTML and PDF versions of the Article.

