# Supplementary material for: Fast modal decomposition for optical fibers using digital holography
Source: Sci Rep. 2017 Jul 26;7:6556. doi: 10.1038/s41598-017-06974-7 (PMC5529422; doi:10.1038/s41598-017-06974-7)
Supplement: Supplementary file 1 — Supplementary Information [file 41598_2017_6974_MOESM1_ESM.pdf]

Supplementary Information

Fast modal decomposition for optical fibers using digital holography

Meng Lyu[1,2], Zhiquan Lin[1,2], Guowei Li[1,2], and Guohai Situ[1,2,*]

[1] Shanghai Institute of Optics and Fine Mechanics, Chinese Academy of Sciences, Shanghai 201800, China

[2] University of the Chinese Academy of Sciences, Beijing 100049, China

[*] Email: ghsitu@siom.ac.cn

Effect of the phase residuals to the mode decomposition

To support our argument on this point, we performed numerical simulations and the results are plotted below. We first synthesized an electric fields U_{out} with the modes LP_{110} , LP_{11e} , LP_{120} and LP_{12e} . The intensity and phase are shown in Fig. R1(a) and (b); the modal coefficients and the relative phase of each mode of U_{out} are shown in Fig. R1(c) and (d), respectively.

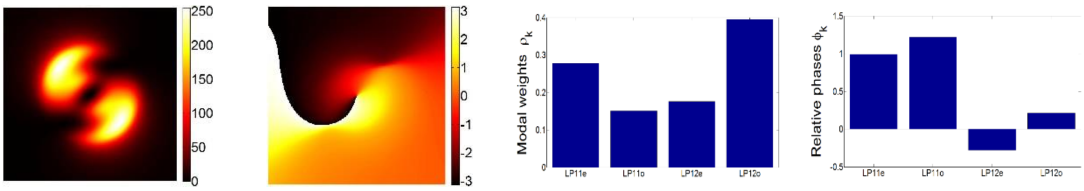

(a) (b) (c) (d)

Fig. R1. The intensity (a) and (b) phase, (c) modal weights and (d) relative phases of the LP mode of U_{out}.

According to our analysis, there are two possible reasons for the fringe in the intensity and the wrapped phase to appear in the reonstructed field. The first one is that the reference beam may not be a perfect plane wave. This happens because of the diffraction. In the first simulation, we added an additional diffracted field to the original electric field U_{out}. The resulting intensity and phase of the new field U_{out1} are shown in Fig. R2(a) and (b). Now we can see that there are fringe patterns appear in the intensity image, and the phase is wrapped. However, by using the proposed MD method, we calculated the modal coefficients and plotted them in Fig. R2(c) and (d). It is clear that the values of both the weights and relative phases of each mode are exactly the same as those plotted in Fig. R1(c) and (d), respectively. By using these coefficients, we synthesized the field U_{syn1}, the intenstity and the phase of which are shown in Fig. R2(e) and (f). And they are exactly equal to Fig. R1(a) and (b) , respectively.

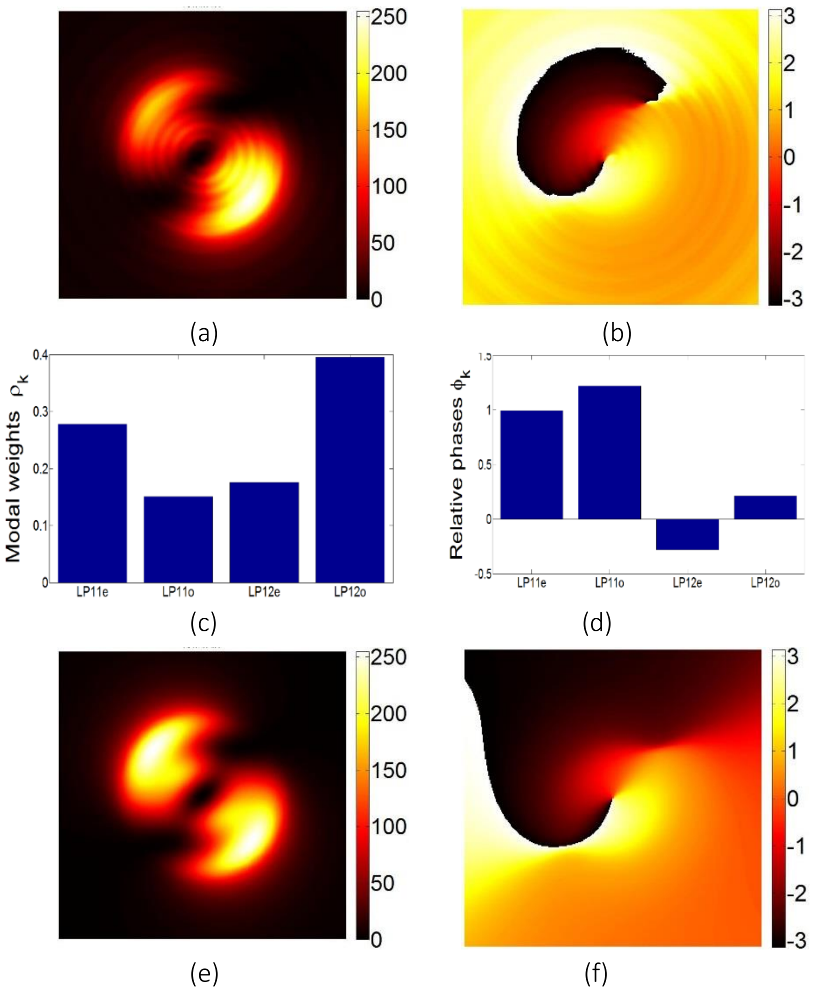

Fig. R2. The intensity (a) and (b) phase, (c) modal weights and (d) relative phases of the LP mode of U_{out1}. By using the decomposed coefficients in (c) and (d), we synthesized the field, the intensity and phase of which are plotted in (e) and (f).

The second one is due to the defocuse of the holographic image. This yields a quadratic phase in U_{out}. We simulated this scenario, and plotted the resulting intensity and phase of the new field U_{out2} in Fig. R3(a) and (b). Again, the intensity has fringe, and the phase is wrapped. However, by using the proposed MD method, we calculated the modal coefficients and plotted them in Fig. R3(c) and (d). It is clear that the values of both the weights and relative phases of each mode are exactly the same as those plotted in Fig. R1(c) and (d), respectively. By using these coefficients, we synthesized the field U_{syn2}, the intenstity and the phase of which are shown in Fig. R3(e) and (f). And, again, they are exactly equal to Fig. R1(a) and (b), , respectively.

The above results demonstrated that slight defocusing of either the reference beam or the signal beam will not affect the result of the mode decomposition. The reason is that the modes propagates in free space independently, and will not interact with one another.

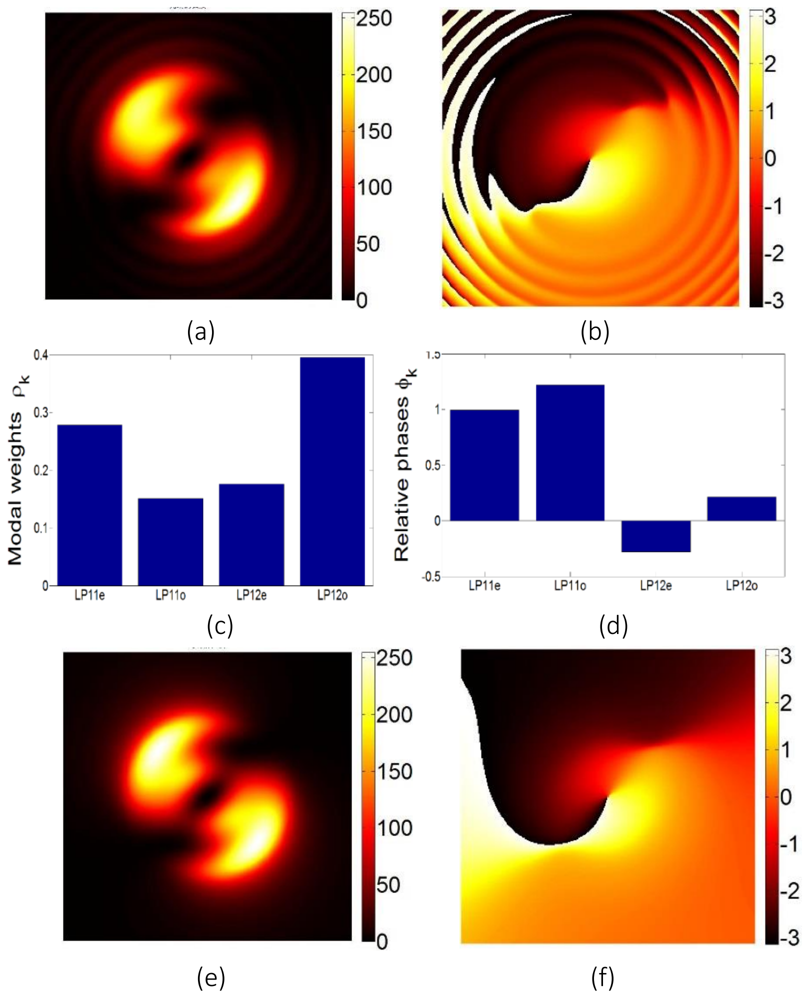

Fig. R3. The intensity (a) and (b) phase, (c) modal weights and (d) relative phases of the LP mode of U_{out2}. By using the decomposed coefficients in (c) and (d), we synthesized the field, the intesnsity and phase of which are plotted in (e) and (f).
